# Novel Protein–Protein Interactions Highlighting the Crosstalk between Hypoplastic Left Heart Syndrome, Ciliopathies and Neurodevelopmental Delays

**DOI:** 10.3390/genes13040627

**Published:** 2022-04-01

**Authors:** Kalyani B. Karunakaran, George C. Gabriel, Narayanaswamy Balakrishnan, Cecilia W. Lo, Madhavi K. Ganapathiraju

**Affiliations:** 1 Supercomputer Education and Research Centre, Indian Institute of Science, Bangalore 560012, India; kalyanik@iisc.ac.in (K.B.K.); balki@iisc.ac.in (N.B.); 2 Department of Developmental Biology, School of Medicine, University of Pittsburgh, Pittsburgh, PA 15201, USA; gcg9@pitt.edu (G.C.G.); cel36@pitt.edu (C.W.L.); 3 Department of Biomedical Informatics, School of Medicine, University of Pittsburgh, Pittsburgh, PA 15206, USA; 4 Intelligent Systems Program, School of Computing and Information, University of Pittsburgh, Pittsburgh, PA 15260, USA

**Keywords:** protein–protein interactions, interactome, congenital heart disease, developmental disorder, hypoplastic left heart syndrome, web application

## Abstract

Hypoplastic left heart syndrome (HLHS) is a severe congenital heart disease (CHD) affecting 1 in 5000 newborns. We constructed the interactome of 74 HLHS-associated genes identified from a large-scale mouse mutagenesis screen, augmenting it with 408 novel protein–protein interactions (PPIs) using our High-Precision Protein–Protein Interaction Prediction (HiPPIP) model. The interactome is available on a webserver with advanced search capabilities. A total of 364 genes including 73 novel interactors were differentially regulated in tissue/iPSC-derived cardiomyocytes of HLHS patients. Novel PPIs facilitated the identification of TOR signaling and endoplasmic reticulum stress modules. We found that 60.5% of the interactome consisted of housekeeping genes that may harbor large-effect mutations and drive HLHS etiology but show limited transmission. Network proximity of diabetes, Alzheimer’s disease, and liver carcinoma-associated genes to HLHS genes suggested a mechanistic basis for their comorbidity with HLHS. Interactome genes showed tissue-specificity for sites of extracardiac anomalies (placenta, liver and brain). The HLHS interactome shared significant overlaps with the interactomes of ciliopathy- and microcephaly-associated genes, with the shared genes enriched for genes involved in intellectual disability and/or developmental delay, and neuronal death pathways, respectively. This supported the increased burden of ciliopathy variants and prevalence of neurological abnormalities observed among HLHS patients with developmental delay and microcephaly, respectively.

## 1. Introduction

Hypoplastic left heart syndrome (HLHS) is a severe form of congenital heart disease (CHD), which is one of the most common birth defects affecting ~1% of live births and a major driver of infant mortality resulting from congenital defects [1]. CHD constitutes structural abnormalities that can affect any cardiac structure including the atria, ventricles, aorta, and pulmonary artery or the valves connecting these chambers. Examples of CHD include atrial and ventricular septal defects, conotruncal defects affecting the ventricular septum and the outflow tract, complex CHD involving disturbance of left–right patterning (e.g., transposition of the great arteries), and valvular defects including inflow (mitral and tricuspid) and outflow (aortic/pulmonic) valves.

CHD classified as left ventricular outflow tract obstructive (LVOTO) lesions comprise a constellation of structural heart defects involving obstruction of flow from the left ventricle (LV). Clinical studies have provided strong evidence of a shared genetic etiology for LVOTO lesions, such as hypoplastic left heart syndrome (HLHS), bicuspid aortic valve (BAV) and coarctation (CoA) [1]. HLHS is a complex CHD, constituting ~1.4 to 3.8% of the CHD cases and estimated to affect 1 in 5000 newborns [2]. It is characterized by underdevelopment of the structures on the left side of the heart, namely, atresia or critical stenosis of the mitral or aortic valves and hypoplasia of the left ventricle, ascending aorta and aortic arch [2]. Until ~30 years ago, infants born with this condition would have died within the first few weeks of life; 23% of the deaths occurring in the first week of life due to cardiac abnormalities have been attributed to HLHS [2,3]. The incidence of HLHS during the fetal stage could be higher. Currently, surgical palliative techniques and improved post-operative care have significantly improved survival, with ~60–70% HLHS neonates surviving for at least 5 years following repair [4,5,6]. Mortality, however, is highest in the first year of life, with 30% of the infants dying or requiring heart transplant before turning one year old. Nevertheless, 90% of those surviving to one year will survive long-term up to 18 years old and beyond [7].

Brain comorbidities such as corpus callosum agenesis, holoprosencephaly, microcephaly and white matter injury have been identified in HLHS neonates, and cognitive, motor and behavioral adverse outcomes such as attention-deficit hyperactivity disorder, learning disabilities, and global developmental delay have been noted among HLHS survivors [8,9]. Given the high mortality and comorbidities associated with HLHS, there is a critical need to investigate the molecular mechanism(s) of disease pathogenesis in HLHS, as only then can therapies be developed to improve outcomes.

In humans, a genetic etiology for HLHS is demonstrated by high familial aggregation of HLHS with other LVOTO defects. Thus, using a statistical framework to calculate genetic effect size, >0.9 heritability was observed for HLHS and >0.7% for HLHS associated with other cardiovascular malformations (*p*-value < 1 × 10^−^^5^) [10]. HLHS is also shown to have a complex multigenic etiology, with clinical studies suggesting a digenic etiology being the most likely [11]. Supporting such complex genetics, a large-scale mutagenesis screen in mice for mutations causing CHD recovered eight mutant lines with HLHS. None shared any genes in common, and none showed Mendelian pattern of inheritance. Together, these findings indicated HLHS has a multigenic etiology and is genetically heterogeneous. Interestingly, the recovery of the HLHS causing mutations in one HLHS mouse line, *Ohia*, confirmed a digenic etiology with mutations in two genes, *Sap130* and *Pcdha9*, shown to cause HLHS [12]. Further supporting a multigenic etiology is the finding that five of the eight HLHS mutant mouse lines had two or more genes in 10 of 14 human chromosome linkage intervals associated with HLHS, with significant enrichment observed when two or more of the mouse HLHS-associated genes were interrogated across these linkage intervals (OR 322.5; CI 24.9–4177.2; *p* = 5.6 × 10^−10^) [13].

CHD-associated de novo mutations in histone-modifying genes such as *KMT2D*, *CHD7*, *KDM5A*, *KDM5B*, *WDR5*, *RNF20*, *UBE2B* and *USP44* were identified in an exome sequencing study conducted with 60 HLHS cases and 264 controls by the Pediatric Cardiac Genomics Consortium (PCGC) [14]. Genome sequencing studies and genome-wide screening by comparative genomic hybridization have identified HLHS-associated variants in cardiomyopathy-associated genes such as *MYBPC3*, *RYR2* and *MYH6* [15], as well as genes associated with mechanotransduction in cardiomyocytes such as *VASP* and *TLN2* [16]. Other genes implicated in HLHS included *RBFOX2*, which mediates RNA metabolism [17], the cardiac transcription factor *PROX1* [18], the endocytic receptor *LRP2* [19], and the transcriptional regulator *POGZ* found in patients with HLHS and developmental delay [20]. However, despite the recovery of genes associated with HLHS, an integrative approach to elucidate their functional consequences is still lacking.

In the current study, we examined HLHS within the mechanistic framework of the protein–protein interaction (PPI) network or protein ‘interactome’. Proteins fuel the cellular machinery, and their interactions reflect the functions that they subserve. This can be informative of disease mechanisms and may also help uncover higher-order relationships in the genetic architecture of complex disorders [21,22,23]. However, only ~145,000 PPIs (25%) out of the estimated ~600,000 PPIs estimated to exist are known from public repositories such as HPRD [24] and BioGRID [25]. Detecting these PPIs using experimental techniques such as the yeast two-hybrid system and co-immunoprecipitation is prohibitively time consuming and expensive. Hence, we have developed a machine learning computational method to predict PPIs called HiPPIP (High-Precision Protein–Protein Interaction Prediction). HiPPIP computes features of protein pairs such as cellular localization, molecular function, biological process membership, genomic location of the gene and gene expression in microarray experiments, and classifies the pairwise features as interacting or non-interacting based on a random forest model [21]. This method has been validated as accurate by computational evaluations [21] and experimental validations [21,26,27]. The novel PPIs predicted using HiPPIP have yielded discoveries with translational impact, including identifying the central role of cilia in CHD [12,21,28]. Here we constructed an ‘HLHS interactome’ with over 400 novel PPIs predicted by HiPPIP and over 1400 known PPIs. We further developed a web resource with the novel PPIs on Wiki-HLHS, an interactive webserver for exploring novel interactions relevant to HLHS proteins or pathways of interest. We demonstrate the utility of the HLHS interactome for discovering higher-order genetic architecture of HLHS based on network analysis, functional enrichment, and transcriptome analyses.

## 2. Materials and Methods

### 2.1. Compilation of HLHS-Associated Genes and Prediction of Novel Interactions

A list of 74 HLHS-associated genes was compiled from HLHS mutant mice, specifically, from 8 independent mouse lines recovered from a large-scale mouse mutagenesis screen [12,28]. This includes all homozygous mutations identified in the 8 HLHS mouse lines and heterozygous mutations also found in the HLHS human linkage intervals. Novel PPIs of the proteins encoded by these genes were predicted using the HiPPIP model that we described in our earlier work [21]. Each HLHS protein (say N1) was paired with each of the other human proteins say, (M1, M2,…, Mn), and each pair was evaluated with the HiPPIP model. The predicted interactions of each of the HLHS proteins were extracted (namely, the pairs whose score is >0.5, a threshold which through computational evaluations and experimental validations was revealed to indicate interacting partners with high confidence). The interactome figures were created using Cytoscape [29].

### 2.2. Identification of Network Modules

Network modules among the HLHS proteins and their interactors were identified using Netbox [30]. Netbox reports modularity and a scaled modularity score, as compared with the modularity observed in 1000 random permutations of the subnetwork. Scaled modularity refers to the standard deviation difference between the observed subnetwork and the mean modularity of the random networks [31].

### 2.3. Functional Enrichment Analysis

Pathway associations of genes in the HLHS interactome were computed using Ingenuity Pathway Analysis (IPA) [32]. Statistical significance of the overlaps between genes in the HLHS interactome and pathways in the Ingenuity Knowledge Base (IKB) was computed with Fisher’s exact test based on hypergeometric distribution. Biological process, cellular component and molecular function (Gene Ontology [33]), pathway (Reactome [34]), disease (OMIM [35] and DisGeNET [36]) and transcription factor target (MSigDB [37]) enrichments were computed using WebGestalt [38]. WebGestalt computes the distribution of genes belonging to a particular functional category in the input list and compares it with the background distribution of genes belonging to this functional category among all the genes that belongs to any functional category in the database selected by the user. Statistical significance of functional category enrichment is computed using Fisher’s exact test and corrected using the Benjamini–Hochberg method for multiple test adjustment. Annotations with FDR-corrected *p*-value < 0.05 were considered significant.

### 2.4. Gene Expression Enrichment Analysis

The enrichment of the HLHS interactome in genes expressed in specific tissues was computed using RNA-sequencing data from 53 postnatal human tissues extracted from GTEx [39]. Two gene sets were compiled for the analysis. The first set contained genes showing high or medium expression (transcripts per million (TPM) ≥ 9) in 53 tissues, provided that they were not housekeeping genes, i.e., genes detected in all the tissues with transcripts per million ≥ 1, as identified in the Human Protein Atlas [40]. The second set contained all the genes that showed high or medium expression in the 53 tissues, irrespective of whether they were housekeeping genes or not. TPM is a metric for quantifying gene expression; it directly measures the relative abundance of transcripts. GMT files served as inputs for the gene over-representation analysis (GSEA) that was conducted based on hypergeometric distribution. Tissue-specificity of the genes in the HLHS interactome was checked using TissueEnrich [41]. The analysis was based on tissue-specific genes compiled from GTEx [39], Human Protein Atlas [40], and Mouse ENCODE [42]. This included ‘tissue-enriched genes’ with at least 5-fold higher mRNA levels in a particular tissue compared to all the other tissues, ‘group-enriched genes’ with at least 5-fold higher mRNA levels in a group of 2–7 tissues, and ‘tissue-enhanced genes’ with at least 5-fold higher mRNA levels in a particular tissue compared to average levels in all tissues.

### 2.5. Network Overlap Analysis

Statistical significance of the overlaps between genes in the HLHS interactome and in the SARS-CoV-2-modulated host protein interactome, the ciliary interactome, the ciliopathy interactome and the microcephaly interactome was computed based on hypergeometric test.

## 3. Results

**Figure 1:**
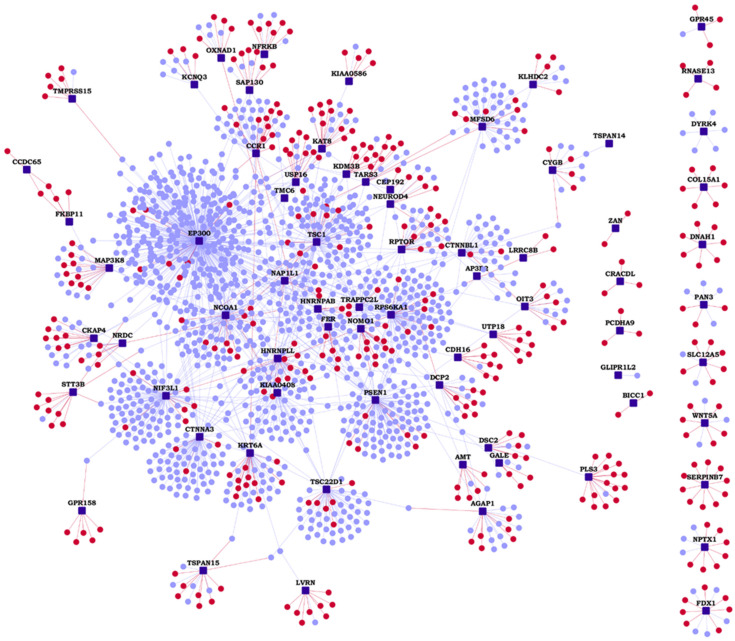
Hypoplastic left heart syndrome (HLHS) protein–protein interactome: Network view of the HLHS interactome is shown as a graph, where genes are shown as nodes and protein–protein interactions (PPIs) as edges connecting the nodes. HLHS-associated genes are shown as dark blue square-shaped nodes, novel interactors and known interactors as red and light blue colored circular nodes, respectively. Red edges are the novel interactions, whereas blue edges are known interactions.

We compiled a list of 74 genes associated with HLHS that were previously identified from eight independent mouse lines with HLHS [12,28]. The protein interactome of these HLHS-associated genes (or ‘HLHS candidate genes’) were assembled by collecting the known protein–protein interactions (PPIs) from the Human Protein Reference Database (HPRD) [24] and the Biological General Repository for Interaction Datasets (BioGRID) [25]. Additionally, we predicted novel PPIs by applying the HiPPIP algorithm, described in our earlier work [21], which is a machine learning algorithm that computes features of protein pairs, such as whether they are co-expressed, or have common biological process or molecular functional annotations, are within the same gene neighborhood, etc., and classifies the feature vector as interacting or non-interacting, using a random forest model. In a recent study, other state-of-the-art algorithms for PPI prediction were extensively evaluated and it was found that none of them reached the superior performance achieved by HiPPIP [43]. Further, seventeen novel PPIs predicted by HiPPIP in relation to other diseases were tested through experimental methods by different research groups, and all those tested were shown to be true PPIs ([21,27] and some unpublished results). Thus, we assembled the HLHS interactome with 1496 previously known PPIs (blue edges in Figure 1) and 408 novel computationally predicted PPIs (red edges in Figure 1), which altogether connected 72 of the 74 HLHS-associated genes with 1248 known interactors and 377 novel interactors (Figure 1 and Supplementary Data File S1). Among the 74 genes, only *WFDC11* and *XKR9* had neither known nor novel PPIs. HiPPIP predicted 644 PPIs of which 236 PPIs were previously known, leaving 408 PPIs to be considered as novel PPIs of the HLHS candidate genes; thus, of the 1496 known PPIs in this interactome, 236 (15.8%) were also predicted by HiPPIP, while 1260 (84.2%) were not (which is as expected, because each method, whether computational or biotechnology based, discovers some of the true PPIs in the interactome, but not all). Four genes identified to be associated with HLHS in independent studies [15,17,18,20] were retrieved as known interactors of our HLHS candidate genes (shown in bold): ***EP300***-*PROX1*, ***TSC1***-*POGZ*, ***HNRNPAB***-*RBFOX2* and ***PSEN1***-*RYR2*.

### 3.1. Wiki-HLHS: A Webserver of HLHS PPIs

To accelerate biomedical discovery, we made the HLHS interactome publicly accessible with the construction of a web application called Wiki-HLHS (http://severus.dbmi.pitt.edu/wiki-HLHS, accessed on 7 January 2022). This webserver has advanced search capabilities, and for each pair of PPIs, there are side-by-side comprehensive Gene Ontology (GO) annotations, and annotations related to diseases, drugs and pathways. Here, a user can query for results such as “show me PPIs where one protein is involved in HLHS and the other is involved in microcephaly”, and then see the results with the functional details of the two proteins side-by-side. This pairwise retrieval of PPIs and their biomedical associations is a unique feature of this web application not available in any other PPI web database. The PPIs and their annotations are also indexed in major search engines such as Google and Bing. A user can browse the genes in the HLHS interactome using list view of HLHS-associated genes. Novel PPIs are shown in a different color in search results.

### 3.2. Identification of Network Modules from the HLHS Interactome

**Figure 2:**
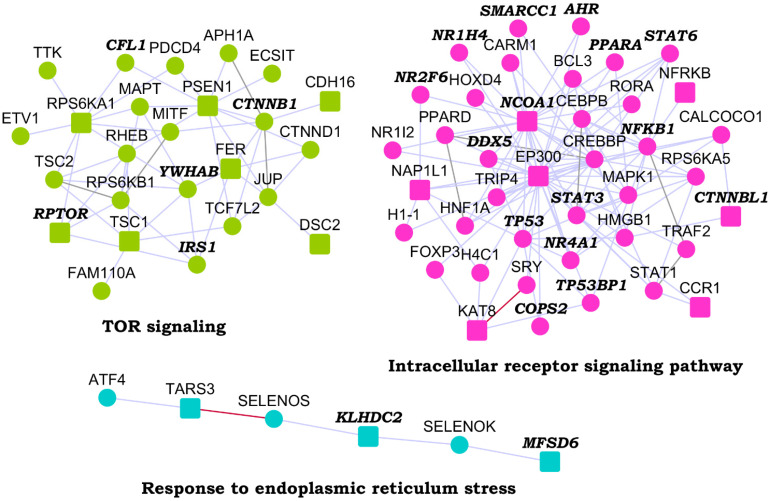
Modules identified from the hypoplastic left heart syndrome (HLHS) interactome: Three modules that were enriched in specific GO biological processes are shown. Within each module, nodes with bold italicized labels depict genes with at least one transcriptomic evidence relevant to HLHS. HLHS-associated genes are shown as square-shaped nodes and novel interactors and known interactors are shown as circular nodes. Red edges are the novel interactions, whereas blue edges are known interactions.

We identified network modules in the HLHS interactome using Netbox [30], starting with the HLHS genes as core genes and adding nodes from the human interactome. The number of edges of node to core genes is statistically significant compared to its degree of interactions in the human interactome. It includes all edges between these nodes and the core genes and identifies highly interconnected modules in this network. Netbox connected 143 proteins (48 HLHS candidate genes and 95 linker proteins) into 19 modules, of which 11 modules had 4 or more nodes each (Supplementary Data File S2). Three modules had statistically significant enrichment of GO biological process terms: *TOR* signaling (*p*-value = 6.97 × 10^−4^, odds ratio = 27.79), response to endoplasmic reticulum (ER) stress (*p*-value = 2.00 × 10^−3^, odds ratio = 55.26) and intracellular receptor signaling pathway (*p*-value = 9.44 × 10^−14^, odds ratio = 20.58) (Figure 2). The novel PPIs facilitated the identification of two functional modules that may be critical to HLHS pathology, namely, TOR signaling and ER stress [44,45].

### 3.3. Functional Enrichment for Human Diseases in the HLHS Interactome

**Figure 3:**
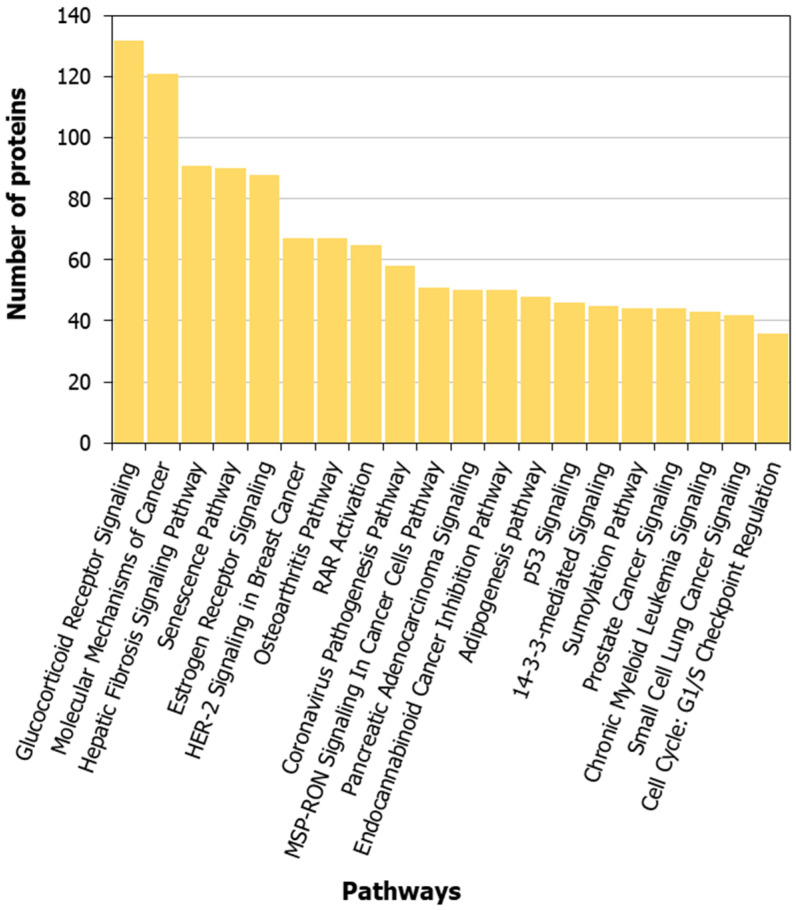
Pathways associated with the hypoplastic left heart syndrome (HLHS) interactome: The number of proteins from the HLHS interactome that are involved in the top 30 pathways most significantly associated with the interactome are shown.

**Figure 4:**
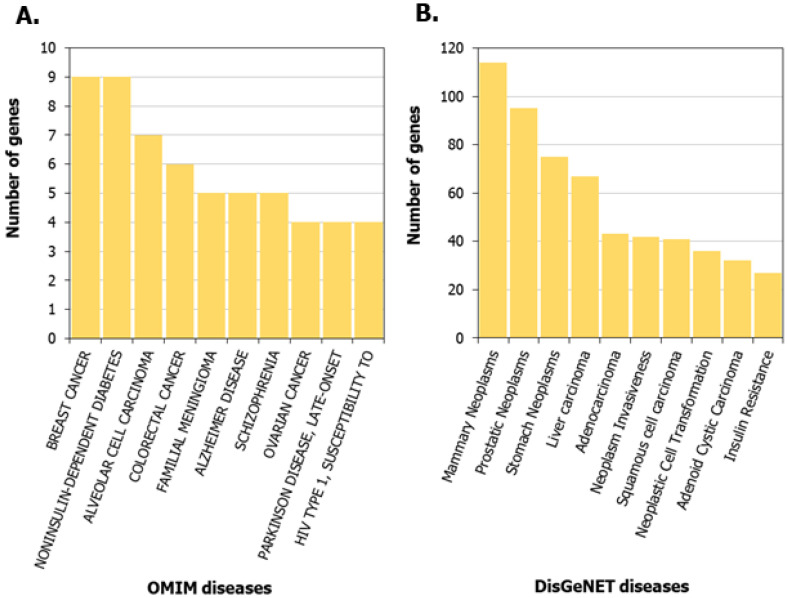
Diseases associated with the hypoplastic left heart syndrome (HLHS) interactome: The number of genes from the HLHS interactome that are involved in the top 10 (**A**) OMIM diseases and (**B**) DisGeNET diseases most significantly associated with the interactome are shown.

**Figure 5:**
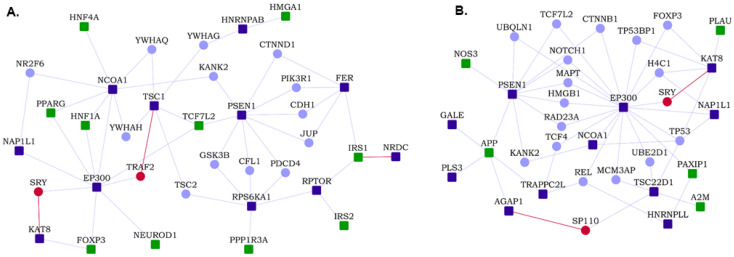
Network proximity of other disease-associated genes to genes associated with hypoplastic left heart syndrome (HLHS): Dark blue square-shaped nodes are HLHS-associated genes and green square-shaped nodes are diabetes-associated genes in (**A**) and Alzheimer’s disease-associated genes in (**B**). Light blue nodes are known interactors and red nodes are novel interactors. Red edges are the novel interactions, whereas blue edges are known interactions.

We compiled the list of pathways for proteins in the HLHS interactome that are associated with the Ingenuity Pathway Analysis suite [32]. Selected pathways that are significantly associated with HLHS are shown in Figure 3 (complete list in Supplementary Data File S3). The Gene Ontology (GO) terms and diseases from OMIM and DisGeNET that are significantly associated with the HLHS interactome at *p*-value < 0.05 are shown in Supplementary Data Files S4–S8. Examination of OMIM-related genes (Figure 4A; Supplementary Data File S7) showed enrichment associated with *non-insulin-dependent diabetes mellitus* (*p*-value = 1.44 × 10^−7^, odds ratio = 14.49) and *insulin-dependent diabetes mellitus* (*p*-value = 0.02, odds ratio = 15.57) in the HLHS interactome, indicating a mechanistic link between HLHS and disease processes related to energy metabolism. Nine diabetes-associated genes that had direct interactions with ten HLHS candidates were responsible for this enrichment, including the novel interaction of the diabetes-associated *IRS1* with the HLHS candidate *NRDC* (Figure 5A; Supplementary Data File S7). Supporting this association, 8.5% of infants born to diabetic mothers have been shown to have congenital heart defects including HLHS, double-outlet right ventricle, truncus arteriosus, transposition of the great arteries and ventricular septal defects [46]. Interestingly, also significantly enriched are genes associated with Alzheimer’s disease (AD) (*p*-value = 1.84 × 10^−5^, odds ratio = 21.23) (Figure 4A; Supplementary Data File S7), with five genes associated with AD exhibiting direct interactions with nine HLHS candidate genes (Figure 5B), supporting a recent study showing increased risk of dementia among patients with congenital heart disease [47]. Finally, examination for enrichment in DisGeNET showed marked enrichment for many different types of cancer, with mammary neoplasms, adenocarcinoma and liver carcinoma being the top three diseases recovered from DisGeNET (Figure 4B; Supplementary Data File S8).

A previous autopsy study showed 43% of HLHS patients have hepatic necrosis [48], and more recent studies have indicated a high incidence of hepatocellular carcinoma among patients having had the Fontan procedure, a third stage surgical palliation that all HLHS patients must undergo [49]. A total of 67 genes associated with liver carcinoma exhibited direct interactions with 28 HLHS candidate genes, including 7 novel interactions (listed in the format HLHS candidate gene-liver carcinoma-associated gene: *NRDC*-*IRS1*, *RPS6KA1*-*CXCL12*, *NIF3L1*-*CXCL12*, *STT3B*-*GNMT*, *ZAN*-*TFPI2*, *COL15A1*-*PDGFB* and *MFSD6*-*STAT1*). We noted that of the top ten diseases recovered from DisGeNET, the top nine are cancer related, but the tenth is “insulin resistance”, further supporting a link between HLHS and diabetes.

### 3.4. GO Biological Process Enrichment and Overlap with HLHS Transcriptome Datasets

Among GO biological processes (Supplementary Data File S4), the most significantly enriched in the HLHS interactome was *covalent chromatin modification* (*p*-value < 1 × 10^−15^, odds ratio = 2.41). This observation was corroborated by the finding that the most significantly enriched GO subcellular locations included *transcription factor complex* (*p*-value < 1 × 10^−6^, odds ratio = 3.14) and *nuclear chromatin* (*p*-value < 1 × 10^−6^, odds ratio = 2.82), and among the molecular functions (Supplementary Data File S6), *DNA-binding transcription activator activity, RNA polymerase II-specific* (*p*-value < 1 × 10^−14^, odds ratio = 3.33) and *transcription coactivator activity* (*p*-value < 1 × 10^−14^, odds ratio = 3.38). Motivated by the enrichment of transcriptional regulatory processes in our interactome and several previous studies suggesting transcriptomic changes associated with HLHS [19,50,51], we further investigated the overlap of the HLHS interactome with four HLHS-related transcriptomic datasets. We studied whether the genes in the HLHS interactome were differentially expressed or alternatively spliced in four different RNA-seq datasets comprising either tissues or cardiomyocytes derived from induced pluripotent stem cells (iPSCs) (Supplementary Data File S9).

Our analysis identified 73 novel interactors (19%) (Table 1) and overall 364 genes (21%) in the HLHS interactome that had one or more transcriptomic association (Supplementary Data File S9). Each of these four RNA-seq datasets showed considerable, albeit statistically non-significant, overlap with the interactome. The datasets include differential expression in cardiomyocytes differentiated from iPSCs of five HLHS patients versus two controls (GSE92447 [51]), yielding 131 genes present in the interactome (odds ratio = 1.09), HLHS-right ventricle versus control-left ventricle/control-right ventricle (GSE23959 [50]), yielding seven genes in the interactome (odds ratio = 2.43), iPSC-derived cardiomyocytes at 25 days from one HLHS proband versus parents yielding 131 genes (odds ratio = 1.01) [19], and genes affected by alternative splicing in HLHS-right ventricle versus control-right ventricle/control-left ventricle (GSE23959 [50]), yielding 136 overlapping genes (odds ratio = 1.02). Though these overlaps are not statistically significant at the systems level, the individual genes and their transcriptomic evidence may provide biologically relevant information about the etiology of HLHS. We did not observe statistically significant overlaps between HLHS transcriptomic data and the HLHS interactome, despite examining all the available RNA-seq datasets. This could be attributed to the tendency of iPSC-derived cell lines to exhibit donor-specific gene expression patterns [52] and sample sizes (in these transcriptomic studies) that are not large enough to capture the genetic heterogeneity of HLHS [53]. Additionally, transcriptomic, proteomic and phenotypic equivalences between these external datasets and the murine-derived gene set used for interactome construction should be interpreted cautiously, unless the biological levels are comprehensively characterized and a clear equivalence of factors is demonstrated in both the species [54]. Nevertheless, it has been shown that the HLHS mouse lines (from which the gene set used for interactome construction was identified) had mutations in two or more genes in 10 of 14 human chromosome intervals associated with HLHS and left ventricle outflow obstruction [12]. In addition, essential features that characterize HLHS, such as hypoplasia of the left ventricle, aorta, and mitral valve, were confirmed in the recovered mouse mutant lines [12].

**Figure 6:**
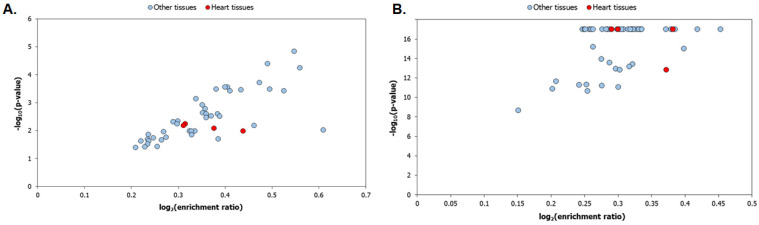
Tissue enrichment of the genes in the hypoplastic left heart syndrome (HLHS) interactome: The tissue enrichment patterns of the HLHS interactome were identified using the gene expression profiles of 53 postnatal human tissues extracted from GTEx. Enrichment was assessed by considering (**A**) any gene that showed high/medium expression in the tissues (transcripts per million (TPM) ≥ 9) and (**B**) any gene that showed high/medium expression in the tissues, except for housekeeping genes (detected in all the tissues with TPM ≥ 1). Statistical significance of tissue enrichment was computed using Fisher’s exact test and corrected using the Benjamini–Hochberg method for multiple test adjustment. It can be observed that the HLHS interactome genes showed a higher statistical significance of enrichment in several tissues (including the heart-related tissues shown as red data points) when housekeeping genes were considered as shown in (**B**) compared to when housekeeping genes were excluded as shown in (**A**), indicating that housekeeping genes could be over-represented in the interactome.

We studied the tissue-specific expression of the HLHS interactome genes using RNA-seq data of 53 postnatal human tissues obtained from GTEx [39], with and without the inclusion of housekeeping genes from the Human Protein Atlas [40]. An expression of more than nine transcripts per million (TPM) is considered high/medium expression. A total of 9634 genes detected in all the tissues with TPM ≥ 1 were considered as housekeeping genes. Statistical significance of the enrichment was computed using Fisher’s exact test and corrected using the Benjamini–Hochberg multiple test adjustment. Compared to when housekeeping genes were excluded, as shown in Figure 6A, the HLHS interactome genes were significantly enriched in several tissues (Figure 6B)—including in heart-related tissues such as the atrial appendage, coronary artery, aorta and the left ventricle—when housekeeping genes were included in the analysis. This could indicate that a large number of genes in the HLHS interactome were housekeeping genes. In line with this, we found that 60.5% (1028 genes) of the interactome was comprised of housekeeping genes, a highly statistically significant over-enrichment (*p*-value = 2.03 × 10^−10^) of 1.14-fold compared to expectations (906 genes). We also noted that the left ventricle showed a lower statistical significance of enrichment compared with the other three heart-related tissues (atrial appendage, coronary artery and aorta).

**Figure 7:**
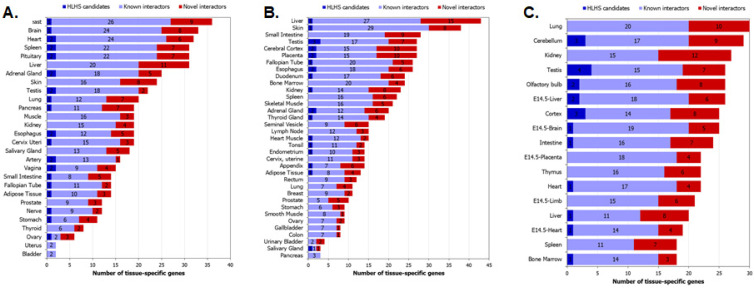
Tissue-specificity of hypoplastic left heart syndrome (HLHS) interactome genes: The graphs show the number of genes from the interactome that exhibit tissue-specificity according to data from (**A**) GTEx, (**B**) Human Protein Atlas and (**C**) mouse ENCODE. The genes show at least 5-fold higher expression in a tissue (‘tissue-enriched’) or a group of 2–7 tissues compared to all the other tissues (‘group-enriched’).

We further employed the TissueEnrich tool to examine the tissue-specificity of the genes in the HLHS interactome based on expression data from GTEx [39], Human Protein Atlas [40] and Mouse ENCODE [42] (Figure 7A–C). Genes with an expression level greater than 1 TPM (transcripts per million) and relative expression at least 5-fold higher in a particular tissue (tissue-enriched) or a group of two to seven tissues (group-enriched) were considered [55]. As expected from an interactome showing an over-enrichment of housekeeping genes, the HLHS interactome did not show any statistically significant tissue-specific enrichment. However, it was noteworthy that six tissues—placenta, skin, liver, lung, brain and testis—showed large overlaps with the HLHS interactome according to data from at least two of the databases and appeared among their lists of top ten tissues (in terms of the number of tissue-specific genes found in the interactome, and not the statistical significance of this overlap) (Figure 7A–C). Ten HLHS candidates had novel PPIs with eleven heart-specific proteins across the three databases (HLHS candidates are shown in bold): ***NFRKB***-*OPCML*, ***NEUROD4***-*IL23A*, ***TSC1***-*PAEP*, ***OIT3***-*PLA2G12B*, ***CDH16***-*CDH5*, ***SLC12A5***-*JPH2*, ***SLC12A5***-*MYL9*, ***DSC2***-*FHOD3*, ***GALE***-*PLA2G5*, ***PLS3***-*NRSN1* and ***TSPAN15***-*ADAMDEC1*.

### 3.5. HLHS and Developmental Delay

Cilia are dynamic projections on cellular surfaces, which detect a wide variety of cues from the environment and transduce signals into the cell to regulate physiological and developmental processes. A genetic screen for recessive CHD-associated mutations had highlighted the role of cilia-transduced cell signaling in CHD [28]. Thirty-four of the sixty-one CHD-associated genes recovered in this screen were cilia-related. To examine for possible ciliary connection to HLHS, we computed the overlap of the HLHS interactome with the interactome of ciliary proteins containing a total of 1665 proteins and 1776 PPIs [56]. The interactomes shared a highly statistically significant overlap (*p*-value = 3.97 × 10^−25^) of 284 genes, and 30% of the overlapping genes were differentially expressed or were affected by alternative splicing in at least one of the four HLHS RNA-seq datasets described in the previous section [19,50,51]. The Reactome pathways *gene expression*, *SUMOylation* and *cell cycle* were enriched among the shared genes by 2.4-fold, 6.4-fold and 3.4-fold, respectively.

**Figure 8:**
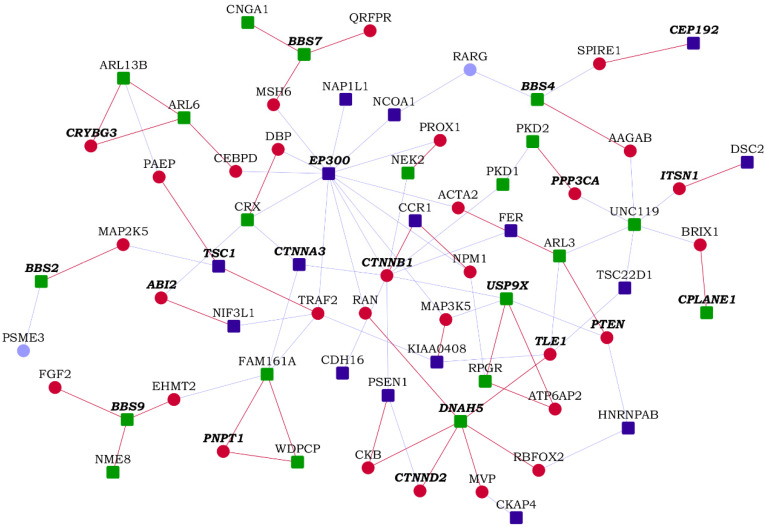
A partial network view of novel protein–protein interactions (PPIs) interconnecting hypoplastic left heart syndrome (HLHS) genes with ciliopathy-associated genes: Genes are shown as nodes and PPIs as edges. As the integrated HLHS and ciliopathy interactome is very large, only a partial view incorporating genes that are associated with intellectual disability and/or developmental delay (ID/DD) and the novel interactors of HLHS-associated genes/ciliopathy-associated genes are shown. Legend—square-shaped dark blue nodes: HLHS-associated genes; square-shaped green nodes: ciliopathy-associated genes; nodes with bold and italicized labels: ID/DD-associated genes; red nodes/edges: novel interactors/interactions; light blue nodes/edges: known interactors/interactions.

Next, we collected a list of 187 genes that have been implicated in 35 ciliopathies from Reiter et al. [57], and assembled its interactome containing 2486 proteins and 3022 interactions. We found that 28% of the HLHS interactome overlapped with 19% of the ciliopathy interactome (473 genes), a highly statistically significant overlap (*p*-value = 1.18 × 10^−58^) with an enrichment ratio of 2-fold compared to expectations (234 genes) (Figure 8 and Supplementary Data File S10). We also found that 67 HLHS-associated genes, 157 ciliopathy-associated genes and 3 genes associated with both (*CCDC65*, *KIAA0586* and *DNAH1*) were connected via 841 intermediate interactors. Eight direct known interactions were found between HLHS candidates and ciliopathy-associated genes (HLHS candidates are shown in bold): ***TSC22D1***-*UNC119*, ***RPTOR***-*CILK1*, ***MFSD6***-*TMEM237*, ***EP300***-*CRX*, ***CTNNA3***-*CRX*, ***CTNNA3***-*FAM161A*, ***NIF3L1***-*NME7* and ***TSC1***-*GLIS2*, and one direct novel interaction, ***RPTOR***-*CCDC40*. We identified the top 30 GO biological processes that were significantly associated with the HLHS and the ciliopathy interactomes and computed the number of genes that were exclusively found in the HLHS/ciliopathy interactomes or shared between the two interactomes in each of these processes (Supplementary Figures S1 and S2). *Regulation of DNA-binding transcription factor activity* was enriched 4-fold among the shared genes between the two interactomes (*p*-value = 3.96 × 10^−12^). Speculating that transcription factor (TF) activity could be a major factor in the crosstalk between HLHS and ciliopathy, we sought to identify the TFs whose target genes were significantly enriched among the genes shared by the HLHS and ciliopathy interactomes. The enrichment analysis was performed using WebGestalt [38] and based on curated TF-target gene sets in MSigDB [37]. The targets of *CREBP1* and *ALX4* showed significant over-enrichment (*p*-values of 2.57 × 10^−4^ and 1.18 × 10^−3^) of 8.68-fold and 38.85-fold (five genes and two genes) compared to expectations. The targets of *CREBP1* found from among the genes shared between the HLHS and ciliopathy interactomes were *EP300*, *PRNP*, *SMAD3*, *SUMO1* and *TBX6*, while the targets of *ALX4* were *JUN* and *TCF7L2*.

HLHS patients having developmental delay as a comorbidity have been shown to have a higher burden of ciliopathy variants compared to HLHS patients without developmental delay, with a summative C-score of 4.05 versus 2.02 (*p*-value of the observed difference < 0.01). Summative C-score is a standardized value used to assess the level of gene disruption in a condition; in this specific study, the C-score of 4.07 was identified as a threshold at which 50% of pathogenic variants and 3% of benign variants were retained [58]. This prompted us to compare the enrichment of genes implicated in developmental delay in the HLHS and ciliopathy interactomes, and specifically among the genes uniquely found in the HLHS/ciliopathy interactomes and those shared between the HLHS and ciliopathy interactomes. A total of 703 genes harboring loss-of-function and missense variants linked to intellectual disability and/or developmental delay (ID/DD) were collected from the Developmental Brain Disorder Gene Database [59]. These ID/DD genes showed an overlap of higher significance with the HLHS interactome (*p*-value = 3.77 × 10^−8^, odds ratio = 1.67) compared with the ciliopathy interactome (*p*-value = 7.61 × 10^−3^, odds ratio = 1.23). Additionally, significant enrichment for ID/DD was shown by genes uniquely found in the HLHS interactome (*p*-value = 7.16 × 10^−6^, odds ratio = 1.65) and genes shared by the HLHS and ciliopathy interactomes (*p*-value = 1.93 × 10^−3^, odds ratio = 1.73) (Figure 8), but not by the genes uniquely found in the ciliopathy interactome. The HLHS candidate *KIAA0586* was associated with ciliopathy as well as ID/DD. Eight other HLHS candidates were linked to ID/DD (i.e., they also harbored ID/DD-associated variants), namely, *TSC1*, *KDM3B*, *CEP192*, *TRAPPC2L*, *EP300*, *CTNNA3*, *KCNQ3* and *AP3B2*. We identified 19 novel PPIs of HLHS candidates with ID/DD genes (HLHS candidates are shown in bold): ***SERPINB7***-*OGDH*, ***SERPINB7***-*PIGN*, ***NOMO1***-*NDE1*, ***NOMO1***-*KCNA1*, ***NCOA1***-*PPP1CB*, ***FER***-*HSD17B4*, ***CKAP4***-*POLR3B*, ***AMT***-*IMPDH2*, ***RPS6KA1***-*ASCC3*, ***STT3B***-*SMARCC1*, ***DSC2***-*ITSN1*, ***AGAP1***-*SLC19A3*, ***OXNAD1***-*SLC6A1*, ***MFSD6***-*HIBCH*, ***KAT8***-*PRRT2*, ***HNRNPAB***-*SYNCRIP*, ***NIF3L1***-*ABI2*, ***PLS3***-*CUL4B* and ***CCR1***-*CTNNB1*.

These results implicate the cilium as a potential focal point for examining HLHS etiology and its comorbid relationships with ciliopathy, intellectual disability and developmental delay.

### 3.6. HLHS and Microcephaly

**Figure 9:**
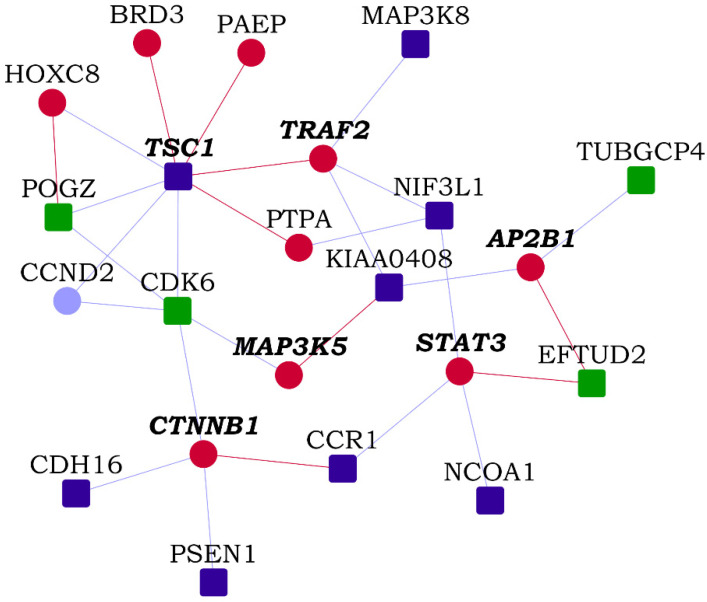
A partial network view of novel protein–protein interactions (PPIs) interconnecting hypoplastic left heart syndrome (HLHS) genes with microcephaly-associated genes: Genes are shown as nodes and PPIs as edges. As the integrated HLHS and microcephaly interactome is very large, only a partial view incorporating genes that are involved in neuronal death processes and the novel interactors of HLHS-associated genes/microcephaly-associated genes are shown. Legend—square-shaped dark blue nodes: HLHS-associated genes; square-shaped green nodes: microcephaly-associated genes; nodes with bold and italicized labels: genes involved in neuron death; red nodes/edges: novel interactors/interactions; light blue nodes/edges: known interactors/interactions.

Severe neurological outcomes such as seizure activity, ischemia, and hemorrhage in HLHS patients are more prevalent with neonatal microcephaly than without (43% versus 4%, *p*-value = 0.02); the prevalence is 33% in HLHS patients with fetal microcephaly (*p*-value = 0.06) [60]. This prompted us to examine their interconnections. A total of 84 genes associated with microcephaly were collected from the MONARCH database [61] and the microcephaly interactome containing 1867 proteins and 2081 interactions was assembled. Sixty-two HLHS candidates were connected to 77 microcephaly genes via 652 intermediate interactors. Five direct known interactions were found between HLHS candidates and microcephaly-associated genes (HLHS candidates are shown in bold): ***TSC1***-*POGZ*, ***TSC1***-*CDK6*, ***CTNNBL1***-*STAMBP*, ***TRAPPC2L***-*TRAPPC6B* and ***TSC22D1***-*QARS1*. We found that 24% of the HLHS interactome overlapped with 22% of the microcephaly interactome (405 genes), a highly statistically significant overlap (*p*-value = 2.39 × 10^−65^) with an enrichment ratio of 2.31-fold compared to expectations (176 genes) (Figure 9 and Supplementary Data File S11). We identified the top 30 GO biological processes that were significantly associated with the HLHS and the microcephaly interactomes and computed the number of genes that were exclusively found in the HLHS/microcephaly interactomes or shared between the two interactomes in each of these processes (Supplementary Figures S3 and S4). Neuron death was enriched 4-fold (32 genes) among the genes common to the HLHS and microcephaly interactomes (*p*-value = 5.13 × 10^−10^). It was also interesting to note that neuron death, neurodegeneration and other related processes and diseases appeared as enriched terms in the HLHS interactome across several functional categories. We extracted a total of 95 genes from the HLHS interactome belonging to these categories, specifically, 5 genes linked to Alzheimer’s disease (OMIM ID: 104300), 4 genes in late-onset Parkinson’s disease (OMIM ID: 168600), 5 genes linked to neurofibrillary degeneration (DisGeNET ID: C0085400), 17 genes in neurodegenerative disorders (DisGeNET ID: C0524851) and 79 genes linked to neuron death (GO ID: 0070997). Two pathways showed high statistical significance and enrichment ratio among these 95 genes, namely, *constitutive signaling by AKT1 E17K in cancer* (*p*-value = 5.94 × 10^−7^, odds ratio = 32.83) and *intrinsic pathway for apoptosis* (*p*-value = 6.94 × 10^−7^, odds ratio = 21.32 folds). A total of 10 novel interactors of HLHS candidates were found among these 95 genes (*ITSN1*, *RHOA*, *NQO1*, *CTNNB1*, *TRAF2*, *MAP3K5*, *PRPH*, *UBQLN2*, *UNC5B* and *FUS*). Five of these novel interactors (*ITSN1*, *RHOA*, *TRAF2*, *CTNNB1* and *UNC5B*) seemed to be involved in *death receptor signaling/apoptosis*, *axon guidance/EPH-Ephrin signaling* and/or *developmental biology*, and their novel PPIs with HLHS candidates were as follows (HLHS candidates are shown in bold): ***DSC2***-*ITSN1*, ***AMT***-*RHOA*, ***CCR1***-*CTNNB1*, ***TSC1***-*TRAF2* and ***OIT3***-*UNC5B*.

Genes in the HLHS interactome linked to neuronal death processes may serve as potential candidates for examining the genetic basis of microcephaly in HLHS patients and the increased prevalence of poor neurological outcomes in these patients. The intriguing link between neurodegenerative processes and HLHS is another result that warrants closer inspection especially in light of the recent finding that adults with congenital heart disease show an increased risk of dementia and early onset dementia, particularly amongst patients with complex lesions [47].

## 4. Discussion

In this study, we adopted a protein interactome analysis approach to study HLHS-associated genes. The interactome analysis framework postulates that diseases develop when PPIs are perturbed by genetic mutations or aberrant expression of genes/proteins, ultimately leading to disrupted cellular functions [62]. Extensive interconnectivity and intraconnectivity of the network components in the PPI network suggest that the effects of such perturbations may spread to other proteins, encoded by genes that do not harbor any disease-associated alterations, through the network of their interactions, posing deeper implications for disease development [62]. In this study, we assembled the HLHS interactome by supplementing previously known protein PPIs with computationally predicted PPIs, which are deemed accurate, and provided valuable insights into etiology through network and enrichment analysis.

We made the PPIs, including the novel PPIs, available on a searchable webserver to enable biologists to study the PPI of their interest (http://severus.dbmi.pitt.edu/wiki-HLHS, accessed on 7 January 2022). Our website provides advanced search capabilities, which allows a user to ask questions that will help generate testable hypotheses around individual PPIs. The full text of the PPIs and their annotations will also be indexed in internet search engines, so that biologists searching in Google, Bing, etc., will find this content. System-level analysis of the interactome with transcriptomic or proteomic data may help to identify its functional landscape. Investigation of individual PPIs will accelerate the understanding of disease biology by several years.

More than 60% of the HLHS interactome, including 51% (38) of the HLHS-associated genes (or ‘core genes’ used for interactome construction), was composed of genes that are constitutively expressed in all the tissues (i.e., housekeeping genes). It has been reported that none of the HLHS-associated mutations harbored by the core genes were shared among the HLHS mutant mouse lines [12]. The preponderance of housekeeping genes among the core genes as well as the HLHS interactome as a whole could explain this genetic heterogeneity. The transmission of mutations in housekeeping genes may be stymied due to their roles in sustaining essential cellular functions, whose perturbation may result in lethality or reduction in reproductive fitness [1]. Although mutations in housekeeping genes are expected to give rise to phenotypes that affect multiple tissues, it is possible that they give rise to cardiac-restricted phenotypes due to complex regulatory influences stemming from epigenetic, epistatic and protein interactions. Further interactome-based investigations driven by this observation (i.e., the enrichment of housekeeping genes in the HLHS interactome) such as those examining vulnerability to network perturbations, and compensatory mechanisms counteracting them, may provide interesting insights.

The HLHS interactome did not show statistically significant enrichment for specific expression in any tissues, as can be expected from the over-enrichment of housekeeping genes in the interactome. However, three out of the six tissues that contained the greatest number of tissue-specific interactome genes have also been documented as sites of extracardiac anomalies in HLHS, namely, the placenta, liver and brain. Specifically, placental abnormalities have been noted in pregnancies that involved fetal HLHS [63]. Increased occurrence of hepatic necrosis has been noted among patients with infantile coarctation of the aorta and HLHS compared with patients having other cardiac defects (38% versus 6%) [64]. The prevalence of brain abnormalities among HLHS neonates and survivors is well-documented [8,9]. The total number of tissue-elevated genes was highly variable among the six tissues that showed the largest overlaps with the HLHS interactome. For example, 2709, 1987, 981, 593, 288 and 197 tissue-elevated genes were found in these tissues, i.e., brain, testis, liver, skin, placenta and lung, respectively, according to the Human Protein Atlas data [40]. Tissues showing a relatively lower number of tissue-elevated genes such as the placenta and lung, as well as those with a higher number of tissue-elevated genes such as the brain, testis, liver and skin, showed overlaps with HLHS interactome. Hence, the overlaps may not have been skewed in relation to the total number of tissue-elevated genes in the tissues. Nevertheless, these statistically non-significant results, which are derived from transcriptomic analysis of the human orthologs of mouse genes, must be interpreted with caution (and after further analysis) in the context of HLHS.

We showed that the interactome of ciliopathy-associated genes shared a significant overlap with the HLHS interactome and that transcription regulation may be over-enriched among these common genes. The targets of transcription factors *CREBP1* and *ALX4* were identified to be significantly enriched among the shared genes. *CREBP1* (also known as *ATF2*) has been shown to regulate the expression of five genes, namely, *EP300* (an HLHS-associated gene sharing a direct interaction with the ciliopathy gene *CRX*), *SUMO1* (a shared interactor having a novel PPI with the ciliopathy-associated gene *MAK* and a known PPI with the HLHS-associated gene *NCOA1*) and three known shared interactors of HLHS and ciliopathy genes (*PRNP*, *SMAD3* and *TBX6*). *ATF2* is involved in cardiomyocyte differentiation [65]. Future studies could concentrate on the role played by *ATF2* and its targets in the shared etiology of ciliopathies and HLHS.

We also showed the preferential enrichment of genes involved in intellectual disability and/or developmental delay (ID/DD) among genes unique to the HLHS interactome and genes shared between the HLHS and ciliopathy interactomes (in comparison with genes unique to the ciliopathy interactome). This finding was in line with the observation of increased ciliopathy variant burden among HLHS patients with developmental delay [58]. Additionally, we provided a list of 19 direct novel PPIs between HLHS-associated and ID/DD genes that may be biochemically validated. For example, *OGDH* is an ID/DD gene that is critical to the tricarboxylic acid cycle and found in the mitochondrial matrix. Loss of *OGDH* has been shown to lead to neurodegeneration [66]. This gene shows high expression in the left ventricle and in brain regions such as the olfactory bulb, hippocampus, cerebellum and pons [67]. This evidence supports *OGDH* as a potential candidate for future studies on the comorbidity of HLHS and ID/DD.

We predicted five direct novel PPIs between HLHS- and microcephaly-associated genes. In addition, genes associated with HLHS and microcephaly share several common interactors that are significantly enriched for neuronal death pathways. This suggests a mechanistic basis for their comorbidity and the increased prevalence of neurological abnormalities among HLHS patients with microcephaly [60]. The over-representation of neurodegenerative disease-associated genes and processes in the HLHS interactome should be investigated, with a focus on the potentially pleiotropic roles of the AKT1-mediated pathways and the intrinsic apoptotic pathway in HLHS and neurodegeneration. The ten direct PPIs between HLHS- and diabetes-associated genes can be used to examine their joint genetic basis and the increased risk of developing HLHS seen among infants born to diabetic mothers [46].

We studied the associations of ciliopathy and microcephaly to the HLHS interactome (on a case-by-case basis) because of their specific relevance to HLHS, namely, (a) increased burden of ciliopathy variants among HLHS patients with developmental delay [58] and (b) increased prevalence of neurological abnormalities among HLHS patients with microcephaly [60]. Further studies may be required to systematically compare the associations of all the phenotypes relevant to HLHS. Nevertheless, we constructed the interactomes of two other disorders that are comorbid with HLHS, namely, chronic kidney disease [68] and cardiovascular disease [69], and compared their overlaps with that exhibited by ciliopathy and microcephaly interactomes. The interactomes of 12 expert-curated chronic kidney disease (CKD)-associated genes and 43 cardiovascular disease (CVD)-associated genes compiled from DisGeNET (with a gene–disease association score > 0.2) showed statistically significant overlaps with the HLHS interactomes (*p*-values of 6.97 × 10^−6^ and 5.47 × 10^−30^). However, fewer genes were shared by the HLHS interactome with CKD (33 genes) and CVD (179 genes) interactomes in comparison with the ciliopathy (473 genes) and microcephaly (405 genes) interactomes. In summary, our study provides evidence for the utility of the HLHS interactome in investigating various HLHS comorbidities and the functional consequences of the genes harboring HLHS-associated mutations. These results will directly inform and catalyze future investigations on the molecular basis of HLHS and biomedical studies seeking to improve clinical interventions in HLHS.

## 5. Conclusions

Knowledge on the exact mechanistic basis of HLHS is limited despite a steady increase in the generation of CHD- and HLHS-related data. In this scenario, the HLHS interactome will serve as a functional landscape to integrate and analyze publicly available HLHS-related multi-omics data and generate new hypotheses that will allow biologists to prioritize pathways and drugs for experimental testing and the developmental of new avenues for therapeutic interventions. To facilitate analysis by both computational and biomedical scientists, the HLHS interactome is being released via an interactive webserver called Wiki-HLHS.

## Figures and Tables

**Table 1:** Novel interactors in the hypoplastic left heart syndrome (HLHS) interactome with biological evidence related to HLHS: The table shows those novel interactors of HLHS-associated genes that have 2 or more HLHS-related biological evidence associated with them. The complete list of biological evidence for all the genes in the interactome can be found in Data File S9.

Gene	Differentially Expressed in Cardiomyocytes from iPSCS of 5 HLHS Patients Versus 2 Controls (GSE92447)	Differentially Expressed in HLHS-Right Ventricle Versus Control-Left Ventricle/Control-Right Ventricle (GSE23959)	Affected by Alternative Splicing in HLHS-Right Ventricle Versus Control-Right Ventricle/Control-Left Ventricle (GSE23959)	Differentially Expressed in 25 Days old iPSC-Derived Cardiomyocytes from 1 HLHS Proband Versus Parents	Total Count
*DBN1*	✓		✓	✓	3
*MYL9*		✓		✓	2
*ASCC3*			✓	✓	2
*CDH5*	✓			✓	2
*CKB*	✓			✓	2
*GART*	✓		✓		2
*PWP1*			✓	✓	2
*TFPI2*	✓			✓	2
*THBS1*	✓			✓	2

## Data Availability

Data are available on the journal website and at http://severus.dbmi.pitt.edu/wiki-HLHS (accessed on 7 January 2022).

## Supplementary Materials

The following are available online at https://www.mdpi.com/article/10.3390/genes13040627/s1: Figure S1: Top 30 Gene Ontology biological processes associated with the hypoplastic left heart syndrome (HLHS) interactome in relation with the ciliopathy interactome, Figure S2: Top 30 Gene Ontology biological processes associated with the ciliopathy interactome in relation with the hypoplastic left heart syndrome (HLHS) interactome, Figure S3: Top 30 Gene Ontology biological processes associated with the hypoplastic left heart syndrome (HLHS) interactome in relation with the microcephaly interactome, Figure S4: Top 30 Gene Ontology biological processes associated with the microcephaly interactome in relation with the hypoplastic left heart syndrome (HLHS) interactome, Data File S1: List of genes from the HLHS interactome, with their labels (HLHS candidate genes, known interactors and novel interactors), and the list of interactions, with their labels (known and novel interactions), Data File S2: List of modules detected in the HLHS interactome, Data File S3: List of all the pathways associated with at least one of the HLHS interactome genes, along with their statistical significance of association (with Bonferroni correction), Data File S4: List of all the Gene Ontology biological process terms significantly associated with the HLHS interactome, Data File S5: List of all the Gene Ontology cellular component terms significantly associated with the HLHS interactome, Data File S6: List of all the Gene Ontology molecular function terms significantly associated with the HLHS interactome, Data File S7: List of all the OMIM diseases significantly associated with the HLHS interactome, Data File S8: List of all the DisGeNET diseases significantly associated with the HLHS interactome, Data File S9: Complete list of HLHS-related biological evidence of genes in the HLHS protein interactome, Data File S10: 473 genes shared between the hypoplastic left heart syndrome (HLHS) interactome and the ciliopathy interactome, and Data File S11: 405 genes shared between the hypoplastic left heart syndrome (HLHS) interactome and the microcephaly interactome.

## References

[1] Zaidi S., Brueckner M. (2017). Genetics and genomics of congenital heart disease. Circ. Res..

[2] Gobergs R., Salputra E., Lubaua I. (2016). Hypoplastic left heart syndrome: A review. Acta Med. Litu..

[3] Šamánek M., Slavík Z., Zbořilová B., Hroboňová V., Voříšková M., Skovranek J. (1989). Prevalence, treatment, and outcome of heart disease in live-born children: A prospective analysis of 91,823 live-born children. Pediatr. Cardiol..

[4] Hamzah M., Othman H.F., Baloglu O., Aly H. (2019). Outcomes of hypoplastic left heart syndrome: Analysis of National Inpatient Sample Database 1998–2004 versus 2005–2014. Eur. J. Pediatr..

[5] D’Udekem Y., Iyengar A.J., Galati J.C., Forsdick V., Weintraub R.G., Wheaton G.R., Bullock A., Justo R.N., Grigg L.E., Sholler G.F. (2014). Redefining expectations of long-term survival after the Fontan procedure: Twenty-five years of follow-up from the entire population of Australia and New Zealand. Circulation.

[6] Alsoufi B., Mori M., Gillespie S., Schlosser B., Slesnick T., Kogon B., Kim D., Sachdeva R., Kanter K. (2015). Impact of patient characteristics and anatomy on results of norwood operation for hypoplastic left heart syndrome. Ann. Thorac. Surg..

[7] Siffel C., Riehle-Colarusso T., Oster M.E., Correa A. (2015). Survival of children with hypoplastic left heart syndrome. Pediatrics.

[8] Marino B.S., Lipkin P.H., Newburger J.W., Peacock G., Gerdes M., Gaynor J.W., Mussatto K.A., Uzark K., Goldberg C.S., Johnson W.H. (2012). Neurodevelopmental outcomes in children with congenital heart disease: Evaluation and management: A scientific statement from the American Heart Association. Circulation.

[9] Hinton R.B., Andelfinger G., Sekar P., Hinton A.C., Gendron R.L., Michelfelder E.C., Robitaille Y., Benson D.W. (2008). Prenatal head growth and white matter injury in hypoplastic left heart syndrome. Pediatr. Res..

[10] Hinton R.B., Martin L.J., Tabangin M.E., Mazwi M.L., Cripe L.H., Benson D.W. (2007). Hypoplastic left heart syndrome is heritable. J. Am. Coll. Cardiol..

[11] McBride K., Pignatelli R., Lewin M., Ho T., Fernbach S., Menesses A., Lam W., Leal S.M., Kaplan N., Schliekelman P. (2005). Inheritance analysis of congenital left ventricular outflow tract obstruction malformations: Segregation, multiplex relative risk, and heritability. Am. J. Med. Genet. Part A.

[12] Liu X., Yagi H., Saeed S., Bais A.S., Gabriel G.C., Chen Z., Peterson K.A., Li Y., Schwartz M.C., Reynolds W.T. (2017). The complex genetics of hypoplastic left heart syndrome. Nat. Genet..

[13] McBride K.L., Zender G.A., Fitzgerald-Butt S.M., Koehler D., Menesses-Diaz A., Fernbach S., Lee K., Towbin J.A., Leal S., Belmont J. (2009). Linkage analysis of left ventricular outflow tract malformations (aortic valve stenosis, coarctation of the aorta, and hypoplastic left heart syndrome). Eur. J. Hum. Genet..

[14] Zaidi S., Choi M., Wakimoto H., Ma L., Jiang J., Overton J.D., Romano-Adesman A., Bjornson R.D., Breitbart R.E., Brown K.K. (2013). De novo mutations in histone-modifying genes in congenital heart disease. Nature.

[15] Theis J.L., Hu J.J., Sundsbak R.S., Evans J.M., Bamlet W.R., Qureshi M.Y., O’Leary P.W., Olson T.M. (2021). Genetic Association Between Hypoplastic Left Heart Syndrome and Cardiomyopathies. Circ. Genom. Precis. Med..

[16] Reuter M.S., Chaturvedi R.R., Liston E., Manshaei R., Aul R.B., Bowdin S., Cohn I., Curtis M., Dhir P., Hayeems R.Z. (2020). The Cardiac Genome Clinic: Implementing genome sequencing in pediatric heart disease. Genet. Med..

[17] Verma S.K., Deshmukh V., Nutter C.A., Jaworski E., Jin W., Wadhwa L., Abata J., Ricci M., Lincoln J., Martin J.F. (2016). Rbfox2 function in RNA metabolism is impaired in hypoplastic left heart syndrome patient hearts. Sci. Rep..

[18] Gill H.K., Parsons S.R., Spalluto C., Davies A.F., Knorz V.J., Burlinson C.E., Ng B.L., Carter N.P., Ogilvie C.M., Wilson D.I. (2009). Separation of the PROX1 gene from upstream conserved elements in a complex inversion/translocation patient with hypoplastic left heart. Eur. J. Hum. Genet..

[19] Theis J.L., Vogler G., Missinato M.A., Li X., Nielsen T., Zeng X.-X.I., Martinez-Fernandez A., Walls S.M., Kervadec A., Kezos J.N. (2020). Patient-specific genomics and cross-species functional analysis implicate LRP2 in hypoplastic left heart syndrome. eLife.

[20] Homsy J., Zaidi S., Shen Y., Ware J.S., Samocha K.E., Karczewski K.J., DePalma S.R., McKean D., Wakimoto H., Gorham J. (2015). De novo mutations in congenital heart disease with neurodevelopmental and other congenital anomalies. Science.

[21] Ganapathiraju M.K., Thahir M., Handen A., Sarkar S.N., Sweet R.A., Nimgaonkar V.L., Loscher C.E., Bauer E.M., Chaparala S. (2016). Schizophrenia interactome with 504 novel protein–protein interactions. NPJ Schizophr..

[22] Lim J., Hao T., Shaw C., Patel A.J., Szabó G., Rual J.-F., Fisk C.J., Li N., Smolyar A., Hill D.E. (2006). A Protein–protein interaction network for human inherited ataxias and disorders of purkinje cell degeneration. Cell.

[23] Sakai Y., Shaw C.A., Dawson B.C., Dugas D.V., Al-Mohtaseb Z., Hill D.E., Zoghbi H.Y. (2011). Protein interactome reveals converging molecular pathways among autism disorders. Sci. Transl. Med..

[24] Prasad T.S.K., Goel R., Kandasamy K., Keerthikumar S., Kumar S., Mathivanan S., Telikicherla D., Raju R., Shafreen B., Venugopal A. (2008). Human protein reference database-2009 update. Nucleic Acids Res..

[25] Stark C., Breitkreutz B.-J., Reguly T., Boucher L., Breitkreutz A., Tyers M. (2006). BioGRID: A general repository for interaction datasets. Nucleic Acids Res..

[26] Zhu J., Zhang Y., Ghosh A., Cuevas R.A., Forero A., Dhar J., Ibsen M.S., Schmid-Burgk J.L., Schmidt T., Ganapathiraju M. (2014). Antiviral activity of human oasl protein is mediated by enhancing signaling of the RIG-I RNA Sensor. Immunity.

[27] Karunakaran K.B., Yanamala N., Boyce G., Becich M.J., Ganapathiraju M.K. (2021). Malignant pleural mesothelioma interactome with 364 novel protein-protein interactions. Cancers.

[28] Li Y., Klena N.T., Gabriel G.C., Liu X., Kim A.J., Lemke K., Chen Y., Chatterjee B., Devine W., Damerla R.R. (2015). Global genetic analysis in mice unveils central role for cilia in congenital heart disease. Nature.

[29] Shannon P., Markiel A., Ozier O., Baliga N.S., Wang J.T., Ramage D., Amin N., Schwikowski B., Ideker T. (2003). Cytoscape: A software environment for integrated models of Biomolecular Interaction Networks. Genome Res..

[30] Cerami E., Demir E., Schultz N., Taylor B.S., Sander C. (2010). Automated network analysis identifies core pathways in glioblastoma. PLoS ONE.

[31] Wang Z., Zhang J. (2007). In search of the biological significance of modular structures in protein networks. PLoS Comput. Biol..

[32] Krämer A., Green J., Pollard J., Tugendreich S. (2014). Causal analysis approaches in Ingenuity Pathway Analysis. Bioinformatics.

[33] Consortium G.O. (2004). The Gene Ontology (GO) database and informatics resource. Nucleic Acids Res..

[34] Croft D., Mundo A.F., Haw R., Orlic-Milacic M., Weiser J., Wu G., Caudy M., Garapati P.V., Gillespie M., Kamdar M.R. (2013). The Reactome pathway knowledgebase. Nucleic Acids Res..

[35] Hamosh A., Scott A.F., Amberger J.S., Bocchini C.A., Valle D., McKusick V.A. (2002). Online Mendelian Inheritance in Man (OMIM), a knowledgebase of human genes and genetic disorders. Nucleic Acids Res..

[36] Piñero J., Bravo À., Queralt-Rosinach N., Gutiérrez-Sacristán A., Deu-Pons J., Centeno E., García-García J., Sanz F., Furlong L.I. (2016). DisGeNET: A comprehensive platform integrating information on human disease-associated genes and variants. Nucleic Acids Res..

[37] Liberzon A., Subramanian A., Pinchback R., Thorvaldsdóttir H., Tamayo P., Mesirov J.P. (2011). Molecular signatures database (MSigDB) 3.0. Bioinformatics.

[38] Liao Y., Wang J., Jaehnig E.J., Shi Z., Zhang B. (2019). WebGestalt 2019: Gene set analysis toolkit with revamped UIs and APIs. Nucleic Acids Res..

[39] Consortium G. (2015). The Genotype-Tissue Expression (GTEx) pilot analysis: Multitissue gene regulation in humans. Science.

[40] Uhlén M., Fagerberg L., Hallström B.M., Lindskog C., Oksvold P., Mardinoglu A., Sivertsson Å., Kampf C., Sjöstedt E., Asplund A. (2015). Tissue-based map of the human proteome. Science.

[41] Jain A., Tuteja G. (2019). TissueEnrich: Tissue-specific gene enrichment analysis. Bioinformatics.

[42] Stamatoyannopoulos J.A., Snyder M., Hardison R., Ren B., Gingeras T., Gilbert D.M., Groudine M., Bender M., Kaul R., Canfield T. (2012). An encyclopedia of mouse DNA elements (Mouse ENCODE). Genome Biol..

[43] Dunham B., Ganapathiraju M.K. (2020). Benchmark Evaluation of Protein–Protein Interaction Prediction Algorithms. Molecules.

[44] Gaber N., Gagliardi M., Patel P., Kinnear C., Zhang C., Chitayat D., Shannon P., Jaeggi E., Tabori U., Keller G. (2013). Fetal Reprogramming and Senescence in Hypoplastic Left Heart Syndrome and in Human Pluripotent Stem Cells during Cardiac Differentiation. Am. J. Pathol..

[45] Xu X., Jin K., Bais A.S., Zhu W., Yagi H., Feinstein T.N., Nguyen P., Criscione J., Liu X., Beutner G. (2022). Uncompensated mitochondrial mediated oxidative stress underlies heart failure in an iPSC-derived model of congenital heart disease. Cell Stem Cell.

[46] Becerra J.E., Khoury M.J., Cordero J.F., Erickson J.D. (1990). Diabetes mellitus during pregnancy and the risks for specific birth defects: A population-based case-control study. Pediatrics.

[47] Bagge C.N., Henderson V.W., Laursen H.B., Adelborg K., Olsen M., Madsen N.L. (2018). Risk of dementia in adults with congenital heart disease: Population-based cohort study. Circulation.

[48] Komatsu H., Inui A., Kishiki K., Kawai H., Yoshio S., Osawa Y., Kanto T., Fujisawa T. (2019). Liver disease secondary to congenital heart disease in children. Expert Rev. Gastroenterol. Hepatol..

[49] Kogiso T., Tokushige K. (2020). Fontan-associated liver disease and hepatocellular carcinoma in adults. Sci. Rep..

[50] Ricci M., Xu Y., Hammond H.L., Willoughby D.A., Nathanson L., Rodríguez M.M., Vatta M., Lipshultz S.E., Lincoln J. (2012). Myocardial alternative RNA splicing and gene expression profiling in early stage hypoplastic left heart syndrome. PLoS ONE.

[51] Yang C., Xu Y., Yu M., Lee D., Alharti S., Hellen N., Shaik N.A., Banaganapalli B., Mohamoud H.S.A., Elango R. (2017). Induced pluripotent stem cell modelling of HLHS underlines the contribution of dysfunctional NOTCH signalling to impaired cardiogenesis. Hum. Mol. Genet..

[52] Carcamo-Orive I., Hoffman G.E., Cundiff P., Beckmann N.D., D’Souza S.L., Knowles J.W., Patel A., Papatsenko D., Abbasi F., Reaven G.M. (2016). Analysis of transcriptional variability in a large human ipsc library reveals genetic and non-genetic determinants of heterogeneity. Cell Stem Cell.

[53] Yagi H., Liu X., Gabriel G.C., Wu Y., Peterson K., Murray S.A., Aronow B.J., Martin L.J., Benson D.W., Lo C.W. (2018). The Genetic Landscape of Hypoplastic Left Heart Syndrome. Pediatr. Cardiol..

[54] Breschi A., Gingeras T.R., Guigó A.B.R. (2017). Comparative transcriptomics in human and mouse. Nat. Rev. Genet..

[55] Fagerberg L., Hallström B.M., Oksvold P., Kampf C., Djureinovic D., Odeberg J., Habuka M., Tahmasebpoor S., Danielsson A., Edlund K. (2014). Analysis of the human tissue-specific expression by genome-wide integration of transcriptomics and antibody-based proteomics. Mol. Cell. Proteom..

[56] Karunakaran K.B., Chaparala S., Lo C.W., Ganapathiraju M.K. (2020). Cilia interactome with predicted protein–protein interactions reveals connections to Alzheimer’s disease, aging and other neuropsychiatric processes. Sci. Rep..

[57] Reiter J.F., Leroux M.R. (2017). Genes and molecular pathways underpinning ciliopathies. Nat. Rev. Mol. Cell Biol..

[58] Geddes G.C., Stamm K., Mitchell M., Mussatto K.A., Tomita-Mitchell A. (2017). Ciliopathy variant burden and developmental delay in children with hypoplastic left heart syndrome. Genet. Med..

[59] Gonzalez-Mantilla A.J., Moreno-De-Luca A., Ledbetter D.H., Martin C.L. (2016). A cross-disorder method to identify novel candidate genes for developmental brain disorders. JAMA Psychiatry.

[60] Hangge P.T., Cnota J.F., Woo J.G., Hinton A.C., Divanovic A.A., Manning P.B., Ittenbach R.F., Hinton R.B. (2013). Microcephaly is associated with early adverse neurologic outcomes in hypoplastic left heart syndrome. Pediatr. Res..

[61] Cacheiro P., Haendel M.A., Smedley D., Consortium I.M.P., Initiative M. (2019). New models for human disease from the International Mouse Phenotyping Consortium. Mamm. Genome.

[62] Barabási A.-L., Gulbahce N., Loscalzo J. (2010). Network medicine: A network-based approach to human disease. Nat. Rev. Genet..

[63] Jones H.N., Olbrych S.K., Smith K.L., Cnota J.F., Habli M., Ramos-Gonzales O., Owens K.J., Hinton A.C., Polzin W.J., Muglia L.J. (2015). Hypoplastic left heart syndrome is associated with structural and vascular placental abnormalities and leptin dysregulation. Placenta.

[64] Weinberg A.G., Bolande R.P. (1970). The liver in congenital heart disease. Effects of infantile coarctation of the aorta and the hypoplastic left heart syndrome in infancy. Am. J. Dis. Child..

[65] Monzen K., Hiroi Y., Kudoh S., Akazawa H., Oka T., Takimoto E., Hayashi D., Hosoda T., Kawabata M., Miyazono K. (2001). Smads, Tak1, and Their Common Target Atf-2 Play a Critical Role in Cardiomyocyte Differentiation. J. Cell Biol..

[66] Yoon W.H., Sandoval H., Nagarkar-Jaiswal S., Jaiswal M., Yamamoto S., Haelterman N.A., Putluri N., Putluri V., Sreekumar A., Tos T. (2017). Loss of nardilysin, a mitochondrial co-chaperone for α-ketoglutarate dehydrogenase, promotes mTORC1 activation and neurodegeneration. Neuron.

[67] Sadakata T., Furuichi T. (2006). Identification and mRNA expression of Ogdh, QP-C, and two predicted genes in the postnatal mouse brain. Neurosci. Lett..

[68] Morgan C., Al-Aklabi M., Guerra G.G. (2015). Chronic kidney disease in congenital heart disease patients: A narrative review of evidence. Can. J. Kidney Health Dis..

[69] Wang T., Chen L., Yang T., Huang P., Wang L., Zhao L., Zhang S., Ye Z., Chen L., Zheng Z. (2019). Congenital heart disease and risk of cardiovascular disease: A meta-analysis of cohort studies. J. Am. Heart Assoc..

