# Supplementary material for: Novel Protein–Protein Interactions Highlighting the Crosstalk between Hypoplastic Left Heart Syndrome, Ciliopathies and Neurodevelopmental Delays
Source: Genes (Basel). 2022 Apr 1;13(4):627. doi: 10.3390/genes13040627 (PMC9032108; doi:10.3390/genes13040627)
Supplement: Supplementary file 1 [file genes-13-00627-s001.zip › Supplementary-Figures.pdf]

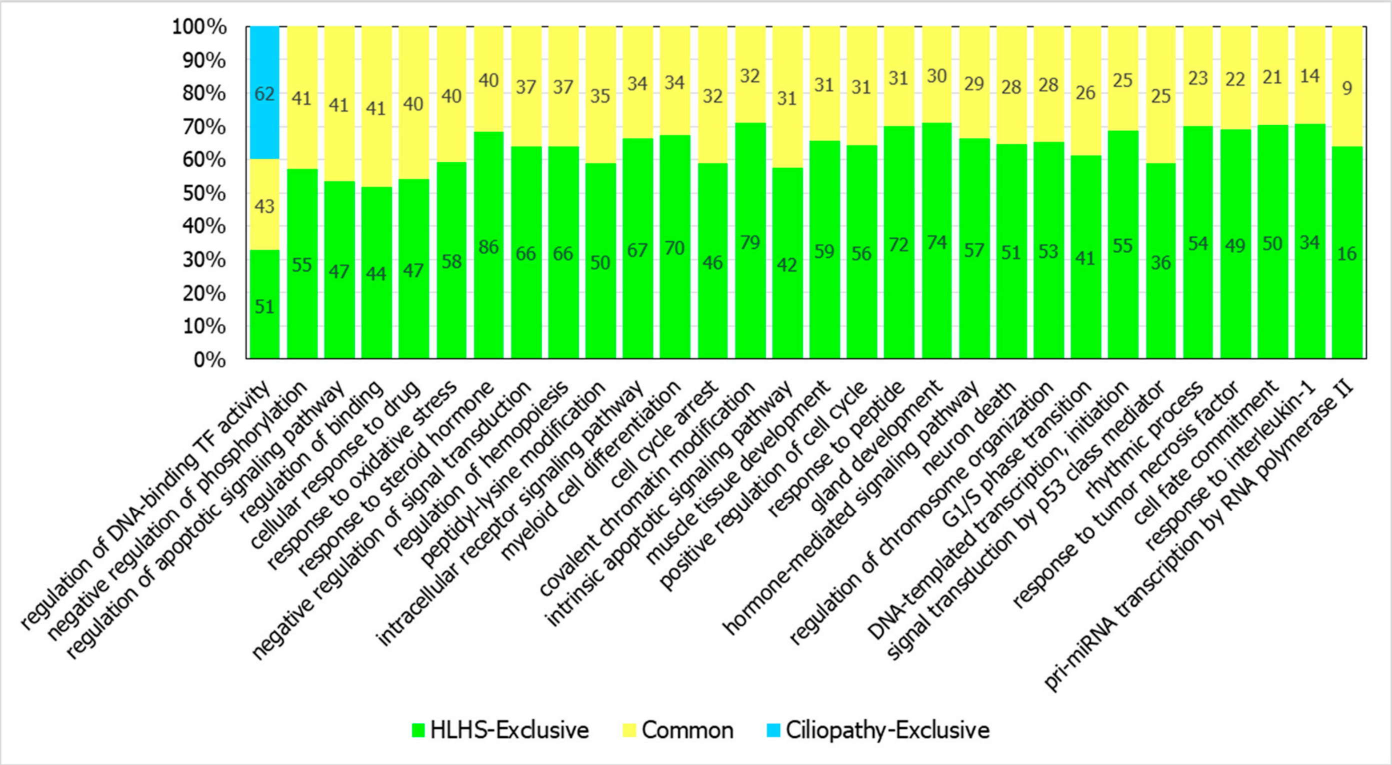

Figure S1. Top-30 Gene Ontology Biological Processes associated with the hypoplastic left heart syndrome (HLHS) interactome in relation with the ciliopathy interactome: Processes associated with the interactome were identified using WebGestalt (web-based gene set analysis toolkit). Statistical significance of GO enrichment was computed using Fisher's exact test, and corrected using the Benjamini-Hochberg method for multiple test adjustment. Shown here are the top-30 processes in the HLHS interactome, along with the number of genes associated exclusively with the HLHS interactome (green), exclusively with the ciliopathy interactome (blue) and common to both (yellow).

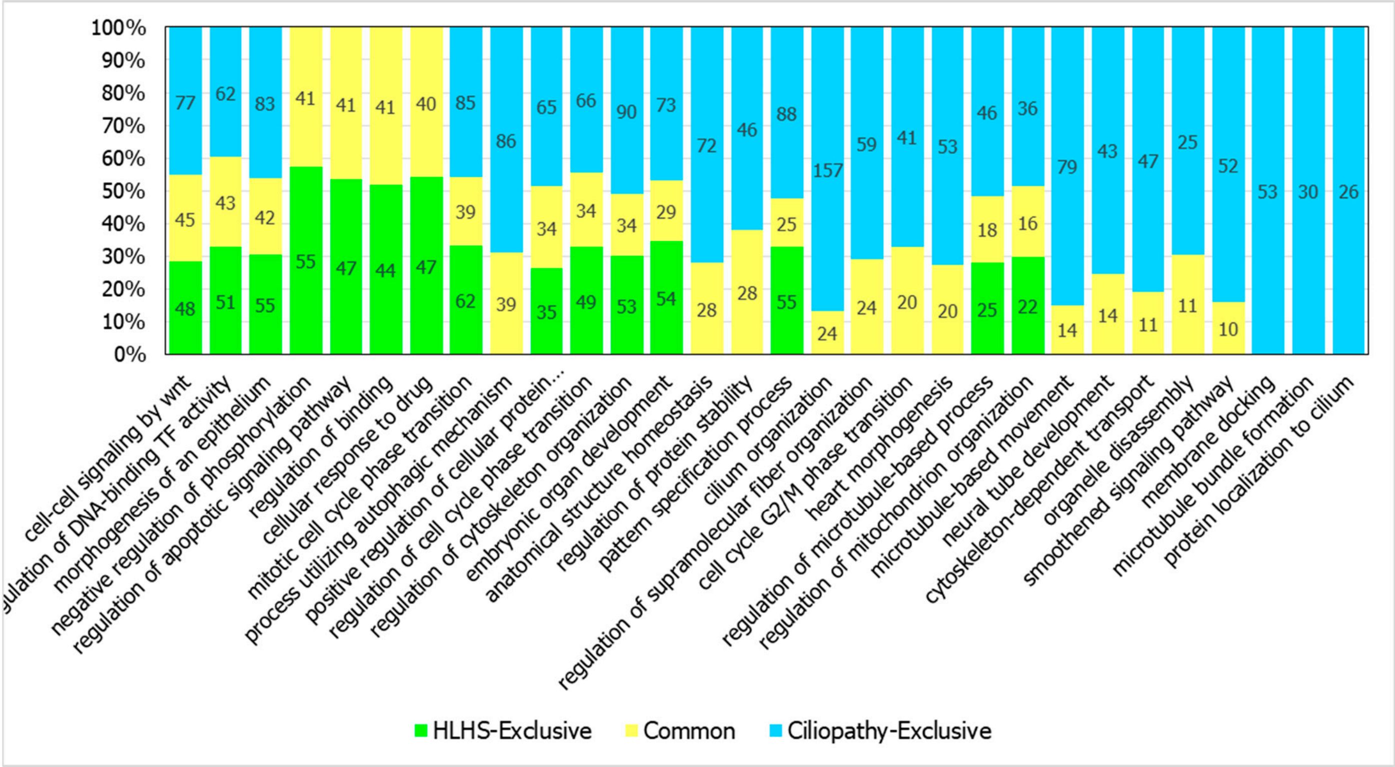

Figure S2. Top-30 Gene Ontology Biological Processes associated with the ciliopathy interactome in relation with the hypoplastic left heart syndrome (HLHS) interactome: Processes associated with the interactome were identified using WebGestalt (web-based gene set analysis toolkit). Statistical significance of GO enrichment was computed using Fisher's exact test, and corrected using the Benjamini-Hochberg method for multiple test adjustment. Shown here are the top-30 processes in the HLHS interactome, along with the number of genes associated exclusively with the HLHS interactome (green), exclusively with the ciliopathy interactome (blue) and common to both (yellow).

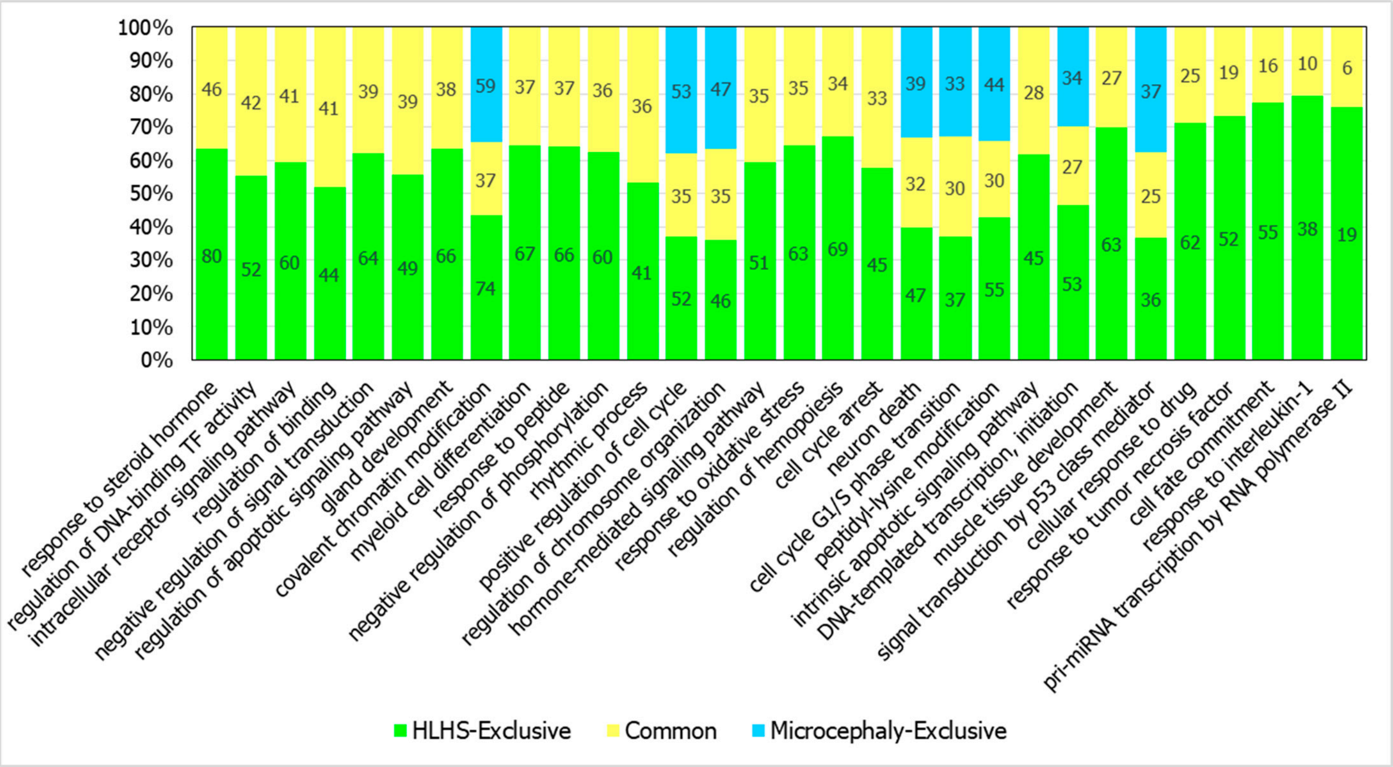

Figure S3. Top-30 Gene Ontology Biological Processes associated with the hypoplastic left heart syndrome (HLHS) interactome in relation with the microcephaly interactome: Processes associated with the interactome were identified using WebGestalt (web-based gene set analysis toolkit). Statistical significance of GO enrichment was computed using Fisher's exact test, and corrected using the Benjamini-Hochberg method for multiple test adjustment. Shown here are the top-30 processes in the HLHS interactome, along with the number of genes associated exclusively with the HLHS interactome (green), exclusively with the microcephaly interactome (blue) and common to both (yellow).

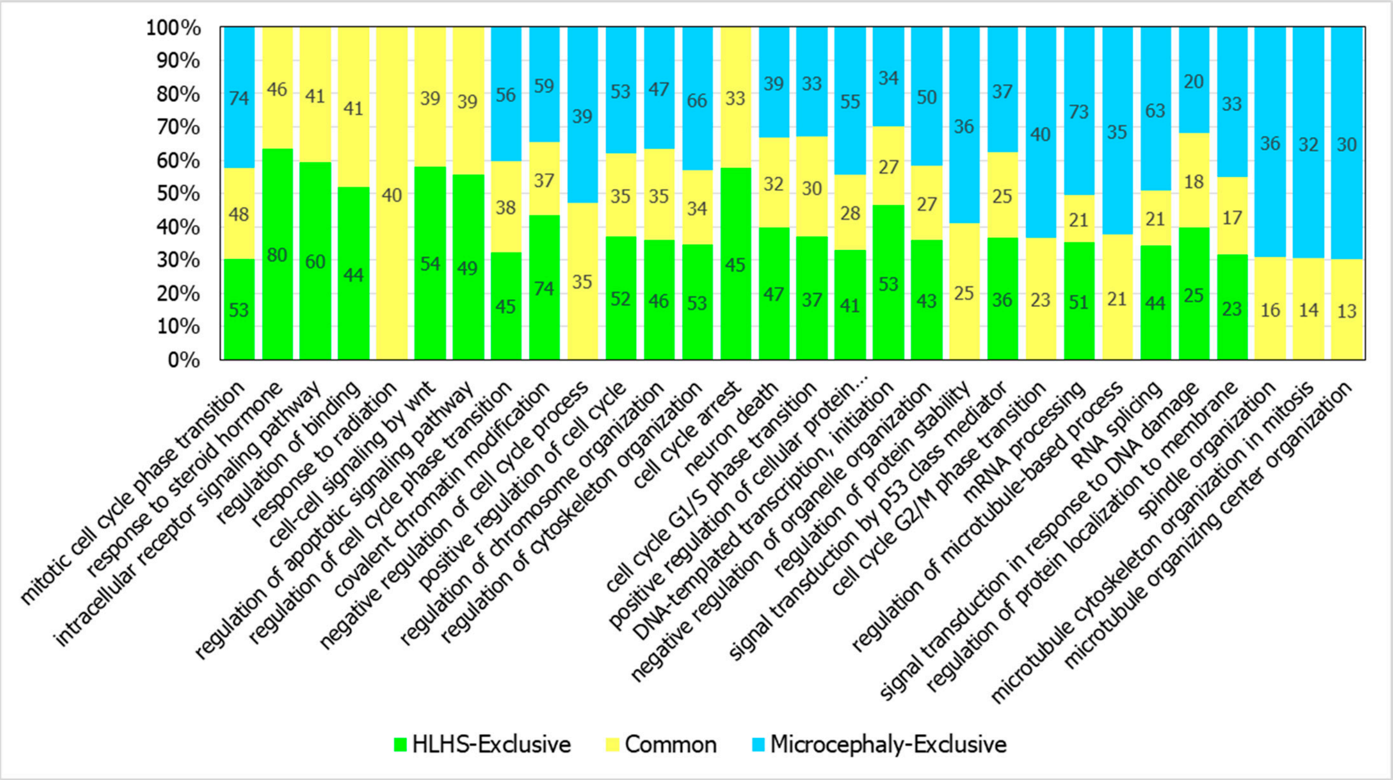

Figure S4. Top-30 Gene Ontology Biological Processes associated with the microcephaly interactome in relation with the hypoplastic left heart syndrome (HLHS) interactome: Processes associated with the interactome were identified using WebGestalt (web-based gene set analysis toolkit). Statistical significance of GO enrichment was computed using Fisher's exact test, and corrected using the Benjamini-Hochberg method for multiple test adjustment. Shown here are the top-30 processes in the HLHS interactome, along with the number of genes associated exclusively with the HLHS interactome (green), exclusively with the microcephaly interactome (blue) and common to both (yellow).
